# Torsion of a Large Appendix Testis Misdiagnosed as Pyocele

**DOI:** 10.1155/2015/430871

**Published:** 2015-03-15

**Authors:** Susanta Meher, Satyajit Rath, Rakesh Sharma, Prakash Kumar Sasmal, Tushar Subhadarshan Mishra

**Affiliations:** Department of General Surgery, All India Institute of Medical Sciences, Sijua, Patrapada, Bhubaneswar, Odisha 751019, India

## Abstract

Torsion of the appendix testis is not an uncommon cause of acute hemiscrotum. It is frequently misdiagnosed as acute epididymitis, orchitis, or torsion of testis. Though conservative management is the treatment of choice for this condition, prompt surgical intervention is warranted when testicular torsion is suspected. We report a case of torsion of a large appendix testis misdiagnosed as pyocele. Emergency exploration of it revealed a large appendix testis with torsion and early features of gangrene. After excision of the appendix testis, the wound was closed with an open drain. The patient had an uneventful and smooth postoperative recovery.

## 1. Introduction

Torsion of the appendix testis is a common cause of scrotal pain in children. It occurs during the prepubertal years (ages 7–14), often precipitated by trauma or exercise [1]. Most of the cases present with unilateral pain and swelling of the scrotum and are frequently misdiagnosed as torsion of testis, epididymitis, or epididymoorchitis. Misdiagnosis of pyocele against torsion of a large appendix testis is an extremely rare event.

## 2. Case Report

**Figure 1:**
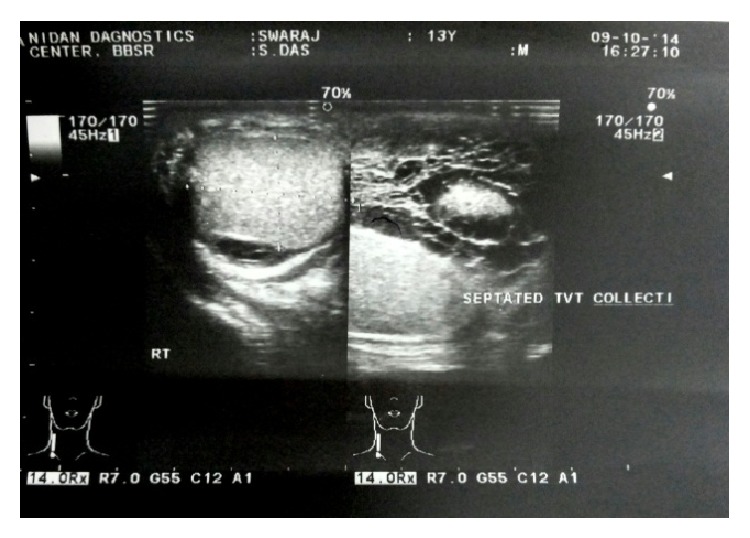
Septated collection.

**Figure 2:**
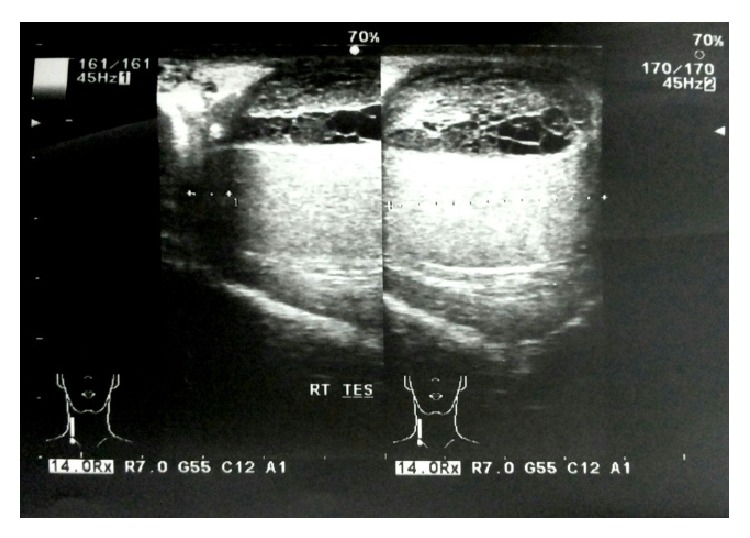
Right testis.

A 13-year-old boy presented to the surgical OPD (outpatient department) with history of a scrotal swelling on right side since 3 years, pain in right hemiscrotum for 10 days, and fever for 3 days. The patient started with a gradually increasing swelling of the right hemiscrotum for 3 years. 10 days before his presentation, he developed pain over the swelling which was dull aching and continuous in nature associated with fever which was mild and continuous. There was no precedent history of any trauma or exercise. On examination the patient had swelling of the right hemiscrotum with edema and erythema of the scrotal skin. Blue dot sign was absent. On palpation local temperature was raised, skin was tender, and testis was not palpable separately. Left side scrotum and testis were normal. On investigation hemoglobin was 12.8 g/dL, total leukocyte count was 12140 per microliter, and other blood parameters were within normal limits. A thick walled septated collection was found in the right tunica vaginalis testis with floating internal debris on ultrasonography (Figure 1). Both testes, mediastinum testis, and the cord structure were normal (Figure 2). Scrotal skin appeared thickened and edematous. Doppler study revealed an enlarged right epididymis with increased vascularity with a normal flow pattern into the right testis. On the basis of clinical findings and investigation a provisional diagnosis of right epididymitis with right pyocele was made.

**Figure 3:**
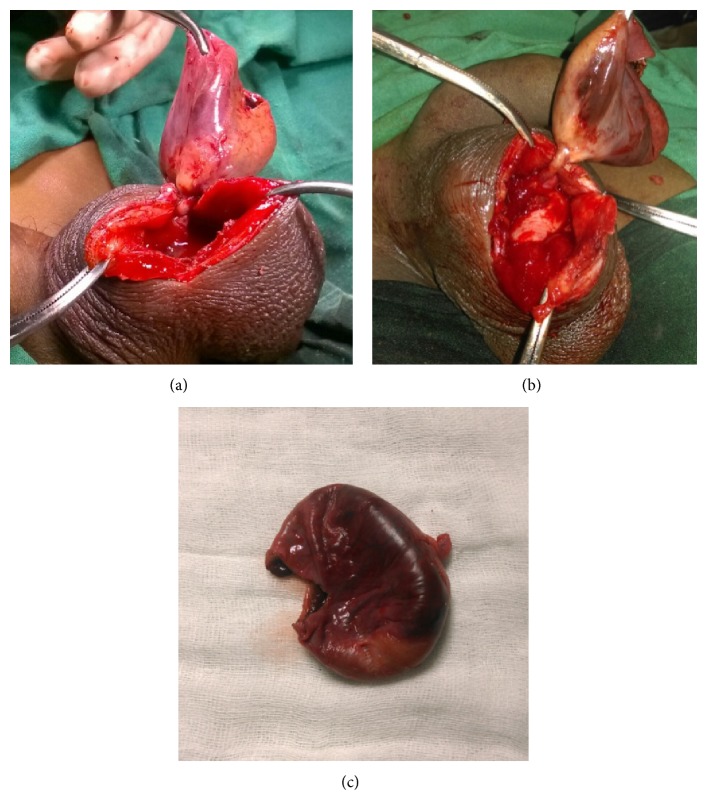
Torsion of appendix testis (a) arising from upper pole of right testis (b) and cyst with gangrenous changes in the wall with a stalk (c).

Emergency exploration was done which revealed a large cyst of approximately 5 × 3.5 × 3 cm in size with a stalk which was arising from the upper pole of the testis (Figure 3(b)). Torsion of the cyst was evident at the stalk (Figure 3(a)). Content of the cyst was gangrenous haemorrhagic fluid with early features of gangrene evident in the wall. Cyst was excised along with the stalk (Figure 3(c)) and sent for histopathological examination. Scrotal wound was closed with an open drain. Recovery was uneventful in the postoperative period and he was discharged after removal of the drain. Histopathology report revealed haemorrhagic infarct with organization and fibroblastic proliferation secondary to torsion without any evidence of neoplastic degeneration. On followup, patient is doing well.

## 3. Discussion

The appendix of testis otherwise known as hydatid of Morgagni is a remnant of upper portion of the paramesonephric duct (Müllerian duct), whereas portion of the mesonephric duct, cranial to the testis, forms the appendix of epididymis [2]. In 1913, Ombredanne mentioned the torsion of appendix testis, but the first case report was published in 1922 by Colt [3]. It is a common cause of acute scrotal pain in children and is frequently misdiagnosed as acute epididymitis, epididymoorchitis, or torsion of testis. Amongst the patients presenting with acute scrotum, testicular torsion is the most common diagnosis in the prepubertal male [4]. In a study by Knight and Vassy [5] of acute scrotal pain in 395 boys ranging in age from 30 days to 17 years, the frequencies of diagnoses were testicular torsion (38%), epididymitis or orchitis (31%), and torsion appendix testis (24%).

Torsion of appendix testis usually presents with sudden onset hemiscrotal pain without any systemic symptoms or urinary complaints. Clinically, the scrotum may be swollen and edematous with tenderness limited to the upper pole of the testis. Presence of a paratesticular nodule along with blue dot sign is pathognomonic for the diagnosis of this condition but this is found only in 21% of cases. Blue dot sign with a normally palpable, nontender testis usually excludes torsion of testis clinically but absence of ipsilateral cremasteric reflex is an indication for strong clinical suspicion of a testicular torsion [6].

Ultrasonography with Doppler done early can be diagnostic. Delay in doing the test frequently leads to misdiagnosis of epididymitis or epididymoorchitis due to increased blood flow to the adjacent epididymis and testis with possibly a reactive hydrocele. An oedematous appendix and head of epididymis sometimes give a “Mickey Mouse” appearance in ultrasonography on transverse lie. Doppler sonography reveals normal flow pattern into the testis, with occasional hypervascularity due to reactive inflammation. Doppler study can achieve a sensitivity of 86% and a specificity of 100% in the diagnosis of testicular torsion [7]. Radionuclide imaging with technetium-99m (99mTc) sodium pertechnetate may show a hot-dot sign due to an area of increased tracer uptake [8].

The treatment for this condition is basically conservative with rest, observation, analgesics, and scrotal support. Surgical intervention is indicated when the diagnosis of testicular torsion cannot be ruled out and the symptoms are prolonged and do not resolve spontaneously. The gangrenous appendage can be excised easily through a small scrotal incision, resulting in prompt relief of symptoms.

## 4. Conclusion

Torsion of appendix testis is a common cause of acute scrotal pain, frequently misdiagnosed because of its atypical presentation. The classical finding of torsion of appendix testis with a typical blue dot sign is seen only in few cases. Strong clinical suspicion with a high resolution sonography with Doppler can diagnose this condition but prompt surgical intervention is required in clinically equivocal cases.
